# Improving Social Cognition in People with Schizophrenia with RC2S: Two Single-Case Studies

**DOI:** 10.3389/fpsyt.2016.00066

**Published:** 2016-04-25

**Authors:** Elodie Peyroux, Nicolas Franck

**Affiliations:** ^1^Rehabilitation Department (CL3R), Le Vinatier Hospital, Lyon, France; ^2^UMR 5229, Center of Cognitive Neurosciences, CNRS, Lyon, France; ^3^University Claude Bernard, University of Lyon, Lyon, France

**Keywords:** social cognition, schizophrenia, cognitive remediation, simulation techniques, single-case design

## Abstract

Difficulties in social interactions are a central characteristic of people with schizophrenia, and can be partly explained by impairments of social cognitive processes. New strategies of cognitive remediation have been recently developed to target these deficits. The RC2S therapy is an individualized and partly computerized program through which patients practice social interactions and develop social cognitive abilities with simulation techniques in a realistic environment. Here, we present the results of two case-studies involving two patients with schizophrenia presenting with specific profiles of impaired social cognition. Each patient completed three baseline sessions, 14 treatment sessions, and 3 follow-up sessions at the end of the therapy – and for 1 patient, another 3 sessions 9 months later. We used a multiple baseline design to assess specific components of social cognition according to the patients’ profiles. Functioning and symptomatology were also assessed at the end of the treatment and 6 months later. Results highlight significant improvements in the targeted social cognitive processes and positive changes in functioning in the long term. The RC2S program seems, thus, to be a new useful program for social cognitive remediation in schizophrenia.

## Introduction

### Social Cognition in Schizophrenia

The most consensual definition of social cognition may be the one proposed by Adolphs (1) as “the ability to construct mental representations of the relation between oneself and others and to use those representations flexibly to guide social behavior.” In social neurosciences, the study of social cognition is at the interface of basic perception processes and behavior in the social world, and includes research in several domains, from social psychology to cognitive sciences (2). In psychiatry, social cognition has been studied since the 1990s in populations with social dysfunction as a central characteristic and diagnostic criterion, such as people with schizophrenia and related disorders. In this disease, impairments have been highlighted at all levels of social processes, from basic perception to social functioning. Social cognitive deficits are now defined as a central characteristic of schizophrenia by the American Psychiatric Association (3).

In people with schizophrenia, social cognitive impairments are a core feature of the disease (4). According to renown researchers, three to five social cognitive processes are usually altered in schizophrenia (5–8): (a) *emotional processing*, or the ability to identify emotions through facial expressions, gestures, and tone of voice; (b) *Theory of Mind* (ToM), defined as “the ability to attribute mental states – beliefs, intents, desires, pretending, knowledge, etc. – to oneself and others and to understand that others have beliefs, desires, and intentions that differ from one’s own” (9); (c) *attributional style*, which refers to how people explain the causes of positive and negative events; in schizophrenia, it is the equivalent of “self-serving” and “personalizing bias,” i.e., the tendency to blame others for negative life events rather than share the responsibilities between different sources (10); and (d and e) *social perception and knowledge*, which can be defined as decoding and interpreting social cues from others, taking the social context into account, and being aware of social rules, roles, and goals.

The components of social cognition appear to be related to both symptomatology and functioning in everyday life. The links between cognitive impairments and symptoms in schizophrenia have been studied since the 1990s. The role of certain cognitive processes in the production of symptoms was first highlighted by Frith’s (11) hypothesis establishing links between self-monitoring and hallucinations. Research on social cognition is more recent. However, it already appears that the alteration of the social processes that allow for the understanding of others’ mental and emotional states may influence both positive and negative symptoms. The positive dimension of schizophrenia has been significantly associated with facial emotion recognition (12) and ToM (13, 14). Attribution bias, and especially hostile attribution bias, has also been correlated with paranoid and persecution delusions (7). Nevertheless, the literature does not always provide consistent results about the links between social cognitive processes and positive symptoms. For the most part, the literature (including recent research) deals with relations between negative symptoms and social cognition and functioning. In this field, specific and significant links between ToM and negative symptoms have been highlighted (15). Other authors have suggested that ToM may be related either to excessive (positive symptoms) or to defective (negative symptoms) attribution of mental states to others (14). Other correlations between negative symptoms and emotional processes have been shown, which suggests that the negative dimension of schizophrenia is closely related to specific processes of social cognition, although both remain two independent constructs (16). To conclude, it seems that social cognitive impairments in schizophrenia, and especially ToM impairments, are directly associated with the severity of negative symptoms (17). According to Couture et al. (18), social cognition and negative symptoms associated with neurocognitive abilities are three pertinent factors to predict the functioning of people with schizophrenia in everyday life.

The social difficulties experienced by people with schizophrenia progressively lead to isolation and relational problems in family, social, and vocational areas. Their quality of life and autonomy are, therefore, strongly impacted, and several studies have looked into the links between social cognition and social functioning. Overall, it seems that social cognition plays a key role in social functioning, professional life, and interpersonal relationships (18, 19). Nevertheless, these links depend on specific social cognitive processes. Couture et al. (19) proposed a conceptual model of relationship between social cognition and functioning. According to them, when people with schizophrenia are facing social interaction, they may first misperceive the emotional expression on the interlocutor’s face without observing additional information from the context. Such misperceptions may then result in drawing the wrong conclusion about the interlocutor’s emotional state. Subsequently, the next processing phase involves finding an explanation as to why the interlocutor is feeling this way. Biases in attributional style, such as personalizing bias, may lead patients to draw wrong conclusions about the cause of their interlocutor’s emotions, since ToM deficits make it difficult for them to walk in someone else’s shoes. This inability to grasp the emotional and social context of the interlocutor’s behavior may lead patients to act inappropriately. According to Couture et al.’s model, impairments (or biases) in social cognition may impact a variety of indices of functional outcomes.

Other meta-analyses were dedicated to the question. Fett et al. (20) highlighted that social cognitive (especially ToM) impairments had major consequences on community functioning, vocational activities, and interpersonal relationships. By comparing the strength of the relations between cognition and functioning, the authors found evidence that social cognition in schizophrenia is more strongly connected to community functioning than neurocognition. Other studies also showed that the quality of life of people with schizophrenia depended more on social cognition (especially ToM) than neurocognition, whereas the opposite was observed in both healthy controls and relatives (21). Accordingly, social cognition (especially ToM) may be a more significant predictor of functional outcomes in schizophrenia.

Several recent modeling studies have more precisely examined the influence of various cognitive factors on social and functional components. Some authors, thus, suggested that social cognition acted as a mediator between neurocognitive functions and community functioning (18, 22, 23). In this context, neurocognition affected functioning through its impact on social cognitive processes. The mediating role of social cognition was also highlighted for professional life (24). Recently, hybrid models of social cognition, acting both as a mediator and an independent contributor, were developed. In those models, neurocognition and social cognition are two independent components that participate separately but closely in daily functioning; social cognition may act as a mediator between some neurocognitive components and functional dimensions (25, 26).

At the moment, one of the most commonly cited models is Schmidt et al.’s (27). The authors gathered 15 studies assessing the mediating role of social cognition between neurocognition and functional status. By applying a structural equation model, they found that neurocognitive impairments in schizophrenia had a negative effect on social cognitive processes, which increased their negative influence on functioning. Schmidt et al.’s model, therefore, confirmed the role of social cognition as a mediator between neurocognition and functioning. Their distal mediation model explained a large proportion of the variance in difficulties observed in functioning – between 7 and 41% with an average of 20%. Nevertheless, other components, and especially social discomfort, support and skills, motivation (28), and negative symptoms (18, 29) were also highlighted as mediating factors between cognition and functioning in schizophrenia.

### Cognitive Remediation of Social Cognition and Advantages of Virtual Environments

In recent years, several cognitive remediation therapies have been developed to treat social cognitive impairments. These interventions use specific learning strategies that overlap with those used in cognitive remediation therapies focusing on neurocognitive processes (30). Strategies include errorless learning (i.e., avoiding the implicit encoding of errors), scaffolding (i.e., minimizing errors by carefully regulating the complexity of learning material), massed practice (i.e., the repetition of tasks to encourage retention and application of skills), information processing strategies (including verbalization, information reduction, breaking, and simplifying the task into various steps), and positive reinforcement (31). The few meta-analyses that have assessed the impact of social cognitive remediation programs highlighted that such therapies showed encouraging results in both patients’ interest and motivation, and social cognitive processes. Kurtz and Richardson’s (32) meta-analysis, including data from 19 studies, highlighted significant effect of social cognitive remediation programs on emotion recognition (moderate-to-large effect) and ToM (small-to-moderate effect). However, most programs did not cause any change on measures of attributional bias. The review of Fiszdon and Reddy (33), including nearly 50 studies and focusing on proximal effects of programs targeting social processes, maintenance of those effects, and generalization to functional outcomes, corroborated previous results. According to them, basic processes of social cognition, such as emotion recognition and social perception, could definitely be improved by cognitive remediation. But these techniques were less successful in remediating more complex, higher-order social cognitive functions. The authors suggested that this might be due to difficulties taking into account or compensate for neurocognitive impairments in schizophrenia, or to limited opportunities for patients to practice skills.

In fact, while the use of computer programs has become very popular in the field of neurocognition, as they offer the possibility to adapt the difficulty level to the specific skills of each patient, to give immediate feedback concerning performance, and to readjust the reinforcement methods used (34), very few has been used in social cognition therapy. This probably arises because it is assumed that interacting with a machine does not involve social skills. This is why, in this field, strategies derived from social skills training, such as role play and exposure to social situations, are used in most programs. It seems, however, that social cognition can be learned directly via computer programs because computerized tasks often provide the opportunity to decompose and control the different processes at play in social interactions, and, thus, to offer a progressive training program regarding difficulty. Moreover, other technologies, such as virtual reality or simulation techniques, are even more applicable.

These techniques expose patients to complex, dynamic, and interactive stimuli and can, thus, serve to remedy cognitive, behavioral, and functional impairments of people with schizophrenia in tasks very close to those found in daily life (35). Indeed, virtual reality and simulation techniques can provide safe and harmless environments where patients can learn social skills without negative repercussions on their real life, such as emotional frustration or a feeling of failure. For these reasons, a number of recent clinical studies have explored the advantage of virtual social skills training, with very positive results, in the field of both autism disorders (36–38) and schizophrenic disorders (39–43). Nevertheless, these programs often focus on general social skills and do not take specific processes of social cognition into account, which are in fact essential to social functioning. In this context, simulation techniques seems highly relevant by allowing to transfer skills, developed in paper-and-pencil tasks, to a realistic and complex environment close to difficulties encountered by patients in daily life. Moreover, traditional programs of social cognitive remediation often focus on relatively simple associations and deductive reasoning involving only one component of social interactions. They use a hierarchical step-by-step model where each process is taught independently and thereby lacks the characteristics of real social situations. We postulate that the RC2S program, by providing an environment similar to the real social world for patients to practice skills in specific social interactions, may be helpful in improving higher-order social cognitive functions and to promote one of the greatest challenges for cognitive remediation: the transfer of acquired abilities to ­everyday life following treatment and the generalization of treatment benefits to other social processes.

### RC2S Program

RC2S is a comprehensive, individualized, and partly computerized social cognitive remediation program (44). It was developed in France through collaboration between the Rehabilitation Department of Le Vinatier Hospital in Lyon and the SBT Company. The treatment lasts 14 weeks at a pace of one 1-to-1 h 30 min session per week, and includes both paper-and-pencil tasks and computerized exercises. The therapy is divided into three main parts.

First, the preparation sessions usually take place over two sessions; the patient and his/her therapist analyze the social cognitive assessment previously performed to determine the patient’s strengths and weaknesses in social cognition. The functional outcomes of social cognitive impairments are examined using a specific questionnaire (Social Cognition-Functional Outcomes Scale or ERF-CS^1^); and information about this cognitive domain are also given thanks to a psychoeducation document (also available in French at the following address: see text footnote 1). Finally, the patient sets two or three concrete objectives designed to improve his/her social functioning by the end of the therapy.

Then, 10 cognitive remediation sessions with the therapist take place once a week for 10 weeks. Each session is divided into four parts: (1) paper-and-pencil tasks for the patient to develop strategies to analyze social situations, focusing first on basic social cognitive processes, such as emotion recognition or social perception, and then on higher order social cognitive functions, such as ToM or attributional style, depending on his/her specific needs and difficulties; (2) a simulation scene in which the patient’s goal is to assist a character named Tom in a particular social situation and guide him by choosing among various propositions of behavioral patterns after each conversation; (3) a review of the situation in which the patient’s choices during the simulation scene are decomposed to focus on specific social components, such as contextual information, tone of voice, gestures, and facial expressions; the therapist can also ask the patient to play the scene again to generate specific mental states in the characters Tom is facing (e.g., anger, envy, or discomfort), identify key components that may appear in real-life interactions, and think about the characters’ possible reactions or intentions; and finally (4) the determination of a home-based task chosen by the patient in collaboration with the therapist and related to the concrete objectives defined at the beginning of the therapy.

Finally, two transfer sessions take place at the end of the social cognitive remediation program. The therapist and the patient review the work done throughout the therapy and assess the achievement of the objectives. These sessions also help the patient transfer the skills acquired during the remediation program to his/her daily life by working on his/her specific difficulties. Thanks to these transfer sessions, the patient can adapt strategies to other social interactions.

This paper reports the RC2S treatment of two patients suffering from schizophrenia. Since cognitive impairments in patients with schizophrenia or related disorders are very diverse, we carried out single-case studies to assess the impact of this new cognitive remediation program. Single-case experimental design has indeed been considered by several authors to assess individualized interventions involving functional issues (45). The two patients enrolled in the studies underwent a complete cognitive, social, and clinical functioning assessment. According to each patient’s profile and objectives, we determined three kinds of baselines before the intervention: targeted component-specific baselines (SBLs); non-SBLs, i.e., measures of neurocognitive processes that should not be affected by the intervention; and IBLs, i.e., measures of social cognitive functions linked to the targeted processes but not directly concerned by the cognitive remediation program. The measures and complete assessments were repeated at the end of the intervention to highlight the impacts of RC2S on social cognitive impairments, and 9 months later (in only one patient) to investigate the possible maintenance of benefits.

## Materials and Methods

### Participants

Damien (Patient A) is a 28-year-old man with a diagnosis of schizophrenia. He presented with difficulties in social relationships and a tendency to withdrawal. He had difficulties communicating with others and exchange during conversations. At the time of recruitment, Damien’s condition was stable after a 5-month hospitalization.

Robin (Patient B) is a 30-year-old man suffering from schizophrenia. He complained about interpersonal relationships, with a difficulty to understand the emotions and mental states of others. He had a tendency to feel hostility from others that affected his vocational integration. Actually, previous relations with his co-workers and superiors were troubled and led to the end of his professional activity as a seasonal worker.

These two single-case studies were carried out in accordance with the Declaration of Helsinki and was approved by the local Ethical Committee (CPP Lyon – Sud Est IV, no. 13/014; AFSAPPS, no. 2013-A00131-44). Written informed consent to take part in the study was received from all participants.

### Assessments

#### Clinical and Functional Assessments

Before starting the RC2S program, clinical, cognitive, and daily life functioning was assessed (the clinical and functional characteristics of patients A and B are summarized in Table 1).

**Table 1:** **Clinical and functional characteristics of patients A and B before the treatment**.

		Patient A	Patient B
PANSS	Total	76	72
	Positive	18	14
	Negative	24	20
	General psychopathology	34	38
SERS	Total	+5	−3
	Negative self-esteem	−34	−34
	Positive self-esteem	+39	+31
WEMWBS	Total	50	43
EAS	Total	42	–
	Personal care	2	–
	Daily life management	12	–
	Resources management	6	–
	External relationships	9	–
	Emotional life and social relationships	13	–

Psychotic symptoms were measured with the positive and negative syndrome scale (PANSS) (46). Before the intervention, symptoms in Damien were not very active, with a moderate score of positive symptoms and a slightly higher score of negative symptoms. Symptoms in Robin were also stable and not very active; both positive and negative symptoms were moderate.

Some aspects of daily functioning were also assessed before the treatment. We measured self-esteem with the Self-Esteem Rating Self (SERS) (47) and mental well-being with the Warwick–Edinburgh Mental Well-Being Scale (WEMWBS) (48). Damien’s self-esteem seemed stable and quite positive, whereas Robin’s was slightly negative. The patients’ mental well-being scores were low and close to the average score of people considering themselves to be in bad health. Finally, the Social Autonomy Scale (*EAS*) (49) was used to assess Damien’s daily and social functioning. Overall, total scores and sub-scores were slightly high, indicating limited autonomy.

#### Cognitive Assessments

Neurocognition and social cognition were assessed in both patients by a different neuropsychologist from the RC2S therapist. Damien’s neurocognitive functioning was affected by weak executive functioning with impaired planning skills [*test des commissions* – revised version; (50)] and mental flexibility [Trail Making Test (TMT); (51)]. Logical deductive reasoning [Matrix reasoning, WAIS IV; (52)], and capacity of abstraction of verbal concepts [Similarities, WAIS IV; (52)] were, however, completely efficient. We noted a slight sensitivity to interference and weak inhibition ability [Stroop (53)]. While assessing attentional processes, we observed that information selection was possible but limited depending on the task. The D2 test (54) highlighted decreased speed of processing when the task was costly. Damien was, however, perfectly able to maintain his attention on the task and appropriately divide attentional resources between several sources of information [Test of Attentional Performance (TAP); (55)]. Finally, Damien had very good resources in both short-term and working memory [Digit span, WAIS IV; (52)].

Neurocognitive functioning of Robin was also globally preserved. His working memory was relatively efficient in both visuospatial [Corsi block-tapping task, MEM III (56)] and verbal modalities [Digit span, Arithmetic and Letter-Number sequencing subtests, WAIS IV; (52)]. Selective and sustained attention was also efficient but speed of processing appeared to be impaired [D2 test (54)]. Robin’s performances in executive functioning were poor, especially mental flexibility [Trail Making Test, TMT; (51)]. Difficulties also showed in the Stroop test (53) but seemed associated with decreased speed of processing.

The evaluation of social cognitive functioning focused on five components: (a) facial emotional processing, (b) affective and cognitive, first- and second-order ToM, including the potential influence of verbalization, (c) attributional style, (d and e) social perception and social knowledge, and (f) empathic abilities (the social cognitive functioning assessments of patients A and B are summarized in Table 2).

**Table 2:** **Social cognitive functioning assessments of patients A and B before the treatment**.

			Patient A		Patient B	
			Raw scores	SD	Raw scores	SD
Facial emotion recognition						
TREF						
		Total percentage of correct answers	66.67	−1.25	62.96	−1.74
		Total detection threshold	60	−1.24	55	−0.81

ToM						
MASC						
		Total score	23	−3	28	−1.65
ToM-15						
		Total score	10	−4.11	11	−3.10
RMET						
		Total score	28	0.84	23	−0.47

Attribution style						
AIHQ						
	Ambiguous situations					
		Hostility score	1.6	1	2.6	3.5
		Attribution of responsibility	1.87	−1.08	3	1.75
		Aggression score	1	−1.5	2	3.5
	Intentional situations					
		Hostility score	–	–	3.2	1.43
		Attribution of responsibility	–	–	4.6	1
		Aggression score	–	–	2.8	2
	Accidental situations					
		Hostility score	–	–	1.4	4
		Attribution of responsibility	–	–	2	0
		Aggression score	–	–	2.4	1.8

Social perception and knowledge						
PerSo						
		Total interpretation score	18/24	–	17/24	–

Empathy						
EQ						
		Total score	39	−0.29	–	–
QCAE						
		Total cognitive	–	–	56/76	–
		Total affective	–	–	39/48	–

Damien’s assessment highlighted poor capacities in identifying and differentiating facial emotions, especially disgust and contempt [TREF (57)]. Damien needed a high intensity of emotion to correctly detect facial expressions. Several ToM measures were performed. MASC-VF [Movie for the Assessment of Social Cognition (58)] showed major and significant deficits. Damien seemed to present with difficulties inferring others’ mental states, i.e., lack of mentalization. Other measures emphasized dissociation between cognitive and affective ToM: the ToM–15 (59), which assesses false-belief understanding, clearly highlighted impaired cognitive ToM, while the Reading the Mind in the Eyes Test (RMET) (60) showed relatively well-preserved affective ToM. Damien’s answers to the Ambiguous Intentions Hostility Questionnaire (AIHQ) (61) on attributional style were relatively appropriate. We did not observe hostility, aggression, or attribution of responsibility bias. Social perception and social knowledge abilities were assessed with a new test (PerSo, GDR 3557) that is currently under validation. Damien’s scores highlighted difficulties using contextual information and selecting relevant cues to understand the social situations depicted in the test. Finally, empathic abilities were assessed using the Empathic Quotient (EQ) (62) and were globally preserved.

Robin’s facial emotion recognition assessment with the TREF (57) highlighted poor capacities in identifying and differentiating facial emotions. These capacities were below average for all emotions except for joy and fear, which were correctly detected even with low emotional intensity. Other emotions were confused even when the level of intensity was the highest. The MASC-VF test (58) for ToM assessment showed important difficulties in understanding others’ intentions, thoughts, and emotions. Robin’s profile seemed to indicate a greater lack of mentalization than tendency to over-interpretation. Moreover, dissociation between cognitive and affective ToM was observed. The ToM-15 (59) clearly revealed impairments, while the RMET (60) showed relatively well-preserved capacities. Robin’s responses to the AIHQ attributional style questionnaire (61) significantly highlighted a hostility bias in accidental and ambiguous situations. In accordance with his complaints in daily life, Robin had a tendency to give hostile explanations about the social situations depicted in the test. Moreover, an aggressive bias was also observed since Robin tended to respond aggressively to intentional and ambiguous situations. Social perception and knowledge abilities (PerSo, GDR 3557) showed that Robin checked images in a fragmented manner, which resulted in difficulties interpreting social situations. Finally, both cognitive and affective empathic abilities assessed using the Questionnaire of Cognitive and Affective Empathy (QCAE) (63) were preserved.

#### Method, Measures, and Statistical Approaches of the Single-Case Studies

Single-case experimental designs represent the intensive and prospective study of an individual, using an *a priori* methodology, which includes systematic observation, manipulation of variables, repeated measurement, and data analysis (64). In contrast to an experimental group design, in which one group is compared with another, participants in a single-subject experiment research provide their own control data for the purpose of comparison in a within-subject, rather than a between-subject, design (65). Single-case designs involve a comparison between experimental time periods, known as *phases*. In our studies, an A–B–A methodological protocol, with (A) a phase with three baseline sessions separated by a 1-week interval to assess the stability of measures; (B) the cognitive remediation therapy phase; and (A) another phase with three baseline sessions at a 1 week interval to assess both the stability of measures and the evolution of performances was used. Damien underwent another phase with three baseline sessions 9 months after the end of the RC2S treatment to analyze the maintenance of benefits, but unfortunately we were not able to do the same with Robin. In this kind of design, the goal is to determine whether a causal or functional relationship exists between a researcher-manipulated independent variable (the introduction of the RC2S treatment) and a meaningful change in the dependent variable (social cognitive measures). The dependent variables are, thus, measured repeatedly across and within the conditions of the study (before and after the treatment). Moreover, in order to control intern validity and to check the absence of confounding variables, additional control measures should be proposed through a multiple baseline design.

In these studies, we selected three kinds of baselines for each patient. We first defined SBLs assessing impaired social cognitive processes, which are the targets of social cognitive remediation therapy, and for which a significant improvement was expected at the end of the therapy so we could prove its effectiveness.

In Damien’s case, two SBLs focusing on ToM were determined: (1) a verbal baseline (SBL1) for which a score out of 12 was calculated in each session, and consisting of short stories derived from Achim et al. (66); the patient answered questions about the mental states of the characters depicted in the stories and (2) a visual baseline (SBL2) consisting of photos extracted from rehabilitation material labeled “Colorcards – what are they thinking” and depicting people in various situations, with balloons representing the protagonists’ thoughts. For each picture, two independent assessors rated a score out of three to obtain a total score out of 15 for each baseline session.

Since Robin’s main social cognitive difficulties lay in attributional style and ToM, two SBLs focusing on these processes were determined. SBL1 aimed to measure causal attributions about positive, negative, or ambiguous events using six sentences for each baseline: two sentences depicting a negative social situation, e.g., “a friend thinks you are stupid”; two sentences depicting a positive social situation, e.g., “a friend tells you that he/she respects you”; and two sentences depicting an ambiguous social situation, e.g., “a friend did not come to the regular appointment.” The sentences were derived from both the Internal, Personal, and Situational Attributions Questionnaire [(IPSAQ) (67); French version by (68)] and the MetaCognitive Training program (MCT) (69). For each sentence, Robin was required to write causal explanations for the situation described. The investigator classified Robin’s propositions into three categories according to the type of attribution: internal (something to do with Robin), personal (something to do with another person or persons), and situational (something to do with the circumstances of chance) attributions. For each sentence, a score from 1 – when Robin suggested causes belonging to only one type of attributions – to 3 – three types of attributions – was calculated. For each baseline session, Robin’s score could, thus, vary from 6 to 18. The second SBL (SBL2) measured ToM by gathering the specific verbal and visual baselines developed for Damien. For each baseline session a total score out of 27 was calculated.

Second, we defined intermediate baselines (IBLs) corresponding to untrained aspects of social functioning that might be impacted by cognitive remediation therapy due to their connections to the targeted processes. The improvement of these measures would evidence the generalization of benefits.

For Damien and Robin, the IBLs targeted two components: (1) facial emotion recognition (IBL1) and (2) social intelligence (IBL2) as defined in Guilford’s (70) work, i.e., the ability to understand others’ behaviors. The emotion recognition baseline consisted of 12 photos extracted from Ekman’s set and eight close-up videos from Baron-Cohen’s “Mind Reading.” The patient had to determine the basic or complex emotions depicted. A score out of 20 was calculated for each baseline session. The “social intelligence” baseline included parts of the Four Factor Test of Social Intelligence developed by O’Sullivan and Guilford (71): expression grouping, missing cartoons, social transactions, and cartoon predictions. A score out of 18 was measured for each baseline session.

Finally, we defined an unspecific baseline (UsBL) measuring a process untargeted by cognitive remediation therapy, for which no evolution was expected by the end of the treatment, so we could evidence treatment specificity. Both patients had to learn 15 abstract words in five trials. Their score was calculated during each baseline session with the sum of the words they were able to recall.

To analyze results, data from all baseline pre-therapy and post-therapy sessions (and 9-month follow-up sessions for Damien) were collected. In the case of Damien, we obtained a 3 × *k* statistical plan, 3 being the three (pre, post, and follow-up) measurement phases and *k* being the baselines with five modalities: SBL/ToM–verbal modality (SBL1), SBL/ToM–visual modality (SBL2), IBL/facial emotion recognition (IBL1), IBL/social intelligence (IBL2), and UsBL. For Robin, we obtained a 2 × *k* statistical plan, 2 being the two (pre- and post-) measurement phases and *k* being the baselines with five modalities: SBL/attributional style (SBL1), SBL/ToM (SBL2), IBL/facial emotion recognition (IBL1), IBL/social intelligence (IBL2), and UsBL.

The analyses were conducted with the *Q*′ (72), which is the equivalent of a non-parametric analysis of variance (ANOVA) based on proportions. The *Q*′ test allows to test the hypothesis of equal proportions and proportion differences in 2 × *k* plan (comparison of two phases in *k* tests or conditions). It derived from Marascuilo’s test (1970) by introducing the Wilson’s variance. In the 2 × *k* design, the magnitude of difference between two proportions is compared in *k* conditions (where a condition may represent a group, a single observer, or even a single test from individual data). The resulting test statistic has a χ^2^ distribution with ν = (*k* − 1) degrees of freedom. The *Q*′ test permits to investigate the main effects and interaction, and a procedure of multiple comparisons can be used to locate statistically significant sources of variance and differences. Since the *Q*′ test allows for the comparison of two phases in *k* tests or conditions (2 × *k* plan), but not a 3 × *k* plan, in the case of Damien we first compared pre- and post-data to assess the impact of the therapy, and post- and follow-up data to assess the maintenance of benefits.

#### Treatment

##### Patient A

The first two sessions allowed Damien to determine the functional outcomes of his social cognitive impairments and objectives for the RC2S therapy. He wanted to enrich his social daily life, be more at ease with others and reconnect with his old friends.

Each cognitive remediation session was divided into three phases: a brief review of the home-based task, paper-and-pencil tasks to develop strategies in specific social situations, and the simulation phase to help transfer strategies into an environment similar to daily life, plus the determination of a new home-based task. At the beginning of the therapy, we worked on strategies deconstructing social situations into microstructures – who, where, when, what – for Damien to take into account their contexts. We then developed a visual scanning of faces strategy to improve his facial emotion recognition abilities. Finally, we worked on ToM reusing the previous strategies. We asked Damien to formulate plausible hypotheses about others’ (emotional and unemotional) mental states based on contextual information, facial, postural, and vocal cues. The same strategies were used during the simulation phase to guide Tom’s behavior and understand the reactions of his interlocutors. Home-based tasks were linked to Damien’s objectives and were intended to favor social interactions, first in family environment and then on the outside to develop friendly relationships.

The last two therapeutic sessions aimed to help transfer strategies into real life. We asked Damien to determine the emotions and mental states of people on the street relying on the previous strategies.

##### Patient B

In accordance with Robin’s complaints, the functional outcomes of social cognitive impairments assessed with the ERF–CS scale (Social Cognition-Functional Outcomes Scale) mainly concerned attributional style. According to both this scale and the social cognitive evaluation, Robin determined two concrete objectives for the therapy. First, he wanted to be less sensitive to negative information during social interactions. To achieve this goal, we planned to focus on attribution bias by developing strategies that helped Robin take into account different interpretations from those he spontaneously made up in his mind. His second objective was to be able to establish contact with others more easily.

Each cognitive remediation session was divided into different phases: a brief review of the home-based task, paper-and-pencil tasks to develop strategies about ambiguous social situations, the simulation phase designed to help transfer strategies into an environment similar to daily life, and finally the determination of a new home-based task. At the beginning of the therapy, we worked on strategies to keep hostile experience at a distance. To achieve this goal, we worked on the links between facial, prosodic and gestural expressions, and internal feelings. Then we helped Robin acquire functional skills to start and maintain a conversation. At the same time, we worked on attribution bias and developed three strategies using both images or video clips and simulation situations from the RC2S program. The first strategy involved thinking about other interpretations of each social interaction using three stereotypical attribution styles: internal, personal, and situational. The second strategy consisted in separating facts from interpretation, and the third was making suggestions and selecting the most probable assumption concerning others’ behaviors. These strategies were also used in home-based tasks and during the last two therapeutic sessions to improve the transfer of strategies to real life.

## Results

### Patient A

At the end of the treatment phase, the interactions between factors were significant [*Q*′(4) = 19.10; *p* = 0.0008]. We observed significant main effects between pre- and post-therapy measures [*Q*′(1) = 19.12; *p* < 0.0001], and between the various baselines [*Q*′(4) = 44.54; *p* < 0.0001]. The results obtained after 9 months showed significant interaction between pre- and follow-up measures [*Q*′(4) = 28.29; *p* < 0.0001], and significant main effects of pre- vs. follow-up measurement phases [*Q*′(1) = 12.19; *p* = 0.0005] and between the various baselines [*Q*′(4) = 48.52; *p* < 0.0001]. No interaction was highlighted between post-therapy and follow-up measures [*Q*′(4) = 2.25; *p* = 0.69]. Multiple comparisons were carried out on each baseline to analyze these results.

We verified that the three measures performed for each baseline at a weekly interval prior to the therapy (pre-therapy), after the treatment (post-therapy), and 9 months later (follow-up) were stable. No significant result was observed. This excluded the possibility that the results might be linked to natural behavioral evolution.

**Figure 1:**
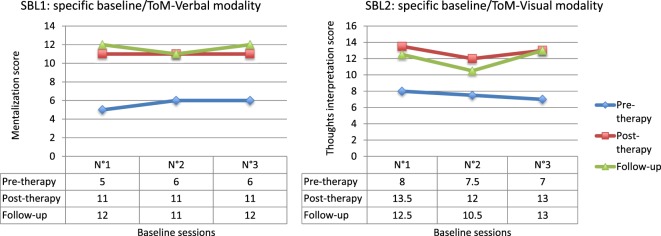
**Results for specific baselines (SBL)**.

The comparison between pre- and post-therapy scores for the SBL1 showed significant improvement (*Q*′ = 4.79; *p* < 0.001). This enhancement appeared stable in the long term, since no significant difference was observed between post-therapy and follow-up measures (*Q*′ = 0.93; *p* = 0.93) and the difference with pre-therapy measures remained significant (*Q*′ = 5.78; *p* < 0.001). The results are similar for the SBL2 (Figure 1). Significant improvements were observed between pre-therapy and both post-therapy (*Q*′ = 4.01; *p* = 0.003) and follow-up scores (*Q*′ = 3.25; *p* = 0.03). No difference was observed between post- and follow-up measures (*Q*′ = 0.71; *p* = 0.97). These results evidence the effectiveness of the RC2S treatment and its maintenance.

**Figure 2:**
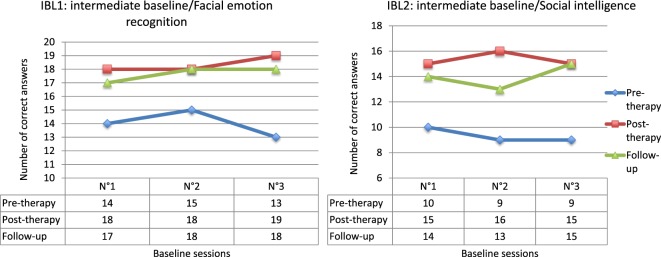
**Results for intermediate baselines (IBL)**.

The results from the emotion recognition IBL (IBL1) highlighted significant improvement at the end of the treatment when comparing pre- and post-therapy scores (*Q*′ = 3.16; *p* = 0.04). This enhancement appeared to be more fragile in the long term, since even if the comparison between post-therapy and follow-up measures was not significant (*Q*′ = 0.60; *p* = 0.99), the difference between pre-therapy and follow-up scores also failed to achieve significance (*Q*′ = 2.57; *p* = 0.16). The results from the social intelligence IBL (IBL2) were similar (Figure 2). Significant enhancement was actually observed between pre- and post-therapy measures (*Q*′ = 3.27; *p* = 0.03), and no difference was highlighted between post-therapy and follow-up measures (*Q*′ = 0.24; *p* = 1). However, the difference between pre-treatment and follow-up measures also failed to achieve significance, even if a trend was observed (*Q*′ = 3.02; *p* = 0.06). These data nevertheless confirm the assumption that benefits tend to generalize to processes close to those targeted by cognitive remediation therapy.

**Figure 3:**
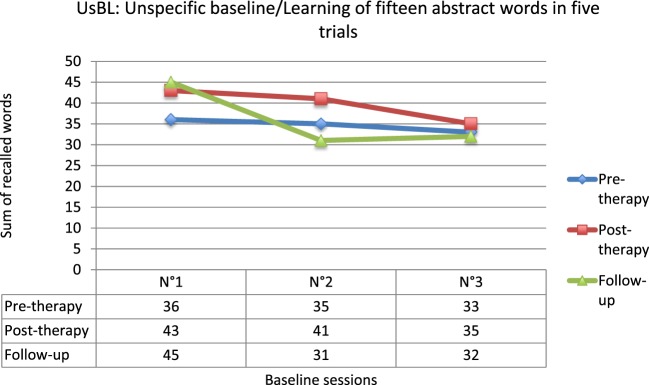
**Results for the unspecific baseline (UsBL)**.

Finally, as we expected it, no evolution of the UsBL score was observed between pre- and post-therapy (*Q*′ = 1.43; *p* = 0.78), nor between pre-therapy and follow-up (*Q*′ = 0.38; *p* = 1) or between post-therapy and follow-up (*Q*′ = 1.05; *p* = 0.90) (Figure 3). These results evidence the specificity of the RC2S program to target social cognitive processes.

### Patient B

At the end of the treatment phase, the interactions between factors were significant [*Q*′(4) = 24.69; *p* = 0.0001]. We observed significant main effects between pre- and post-therapy measures [*Q*′(1) = 20.73; *p* < 0.0001] and between the various baselines [*Q*′(4) = 14.87; *p* = 0.005]. Multiple comparisons were carried out on each baseline to analyze these results.

We verified that the three measures performed for each baseline at a weekly interval prior to the therapy (pre-therapy) and after the treatment (post-therapy) were stable. No significant result was observed. This excluded the possibility that the results might be linked to natural behavioral evolution.

**Figure 4:**
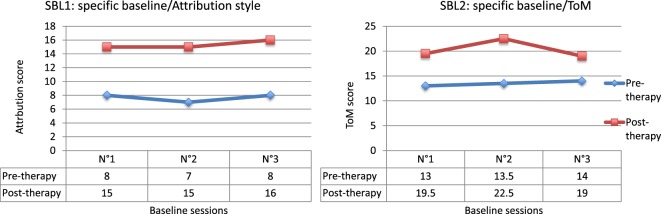
**Results for specific baselines (SBL)**.

The comparison between pre- and post-therapy scores for the attributional style SBL (SBL1) showed significant improvement (*Q*′ = 5.26; *p* < 0.001). The results for the SBL2 are similar (Figure 4). Significant improvements were observed between pre- and post-therapy scores (*Q*′ = 3.52; *p* = 0.015). These results evidence the effectiveness of RC2S on the processes targeted by the treatment.

**Figure 5:**
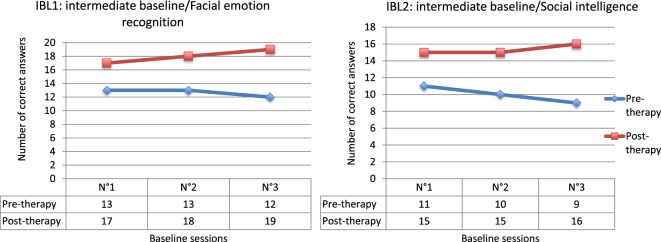
**Results for intermediate baselines (IBL)**.

The results from the IBL1 highlighted significant improvement at the end of the treatment when compared with pre-therapy scores (*Q*′ = 3.69; *p* = 0.009). The results for the IBL2 are similar (Figure 5). Significant enhancement between pre- and post-therapy measures was actually observed (*Q*′ = 3.65; *p* = 0.01). These data confirm the assumption that the benefits generalize to processes close to those targeted by cognitive remediation therapy.

**Figure 6:**
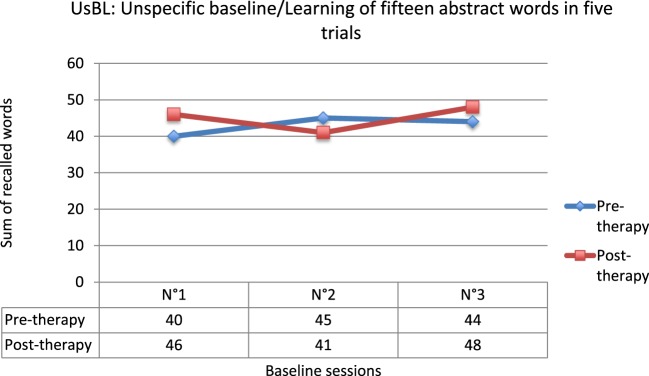
**Results for the unspecific baseline (UsBL)**.

To conclude, no evolution of the UsBL score was observed between pre- and post-therapy (*Q*′ = 0.58; *p* = 0.99), in accordance with our expectations (Figure 6). These results confirm the specificity of the RC2S program.

### Conclusion

The single-case methodology used to assess the influence of the RC2S treatment stressed the positive impact of the therapy on patients’ social cognitive skills. To confirm these observations, the social cognitive assessment performed prior to the treatment was repeated at the end of the program – and 9 months later in Damien’s case. The results are summarized in Table 3.

**Table 3:** **Pre-, post-, and follow-up comparisons of social cognitive assessments for patients A and B**.

			Patient A					Patient B		
			Pre-therapy scores	Post-therapy scores	*p* (pre vs. post)	Follow-up scores	*p* (pre vs. follow-up)	Pre-therapy scores	Post-therapy scores	*p* (pre vs. post)
Facial emotion recognition										
TREF		Total percentage of correct answers	66.67	74.07	ns	81.48	0.08	62.96	72.22	ns
		Total detection threshold	60	49	ns	38	ns	55	52.5	ns

ToM										
MASC		Total score	23	30	0.09	29	ns	28	32	ns
ToM-15		Total score	10	14	<0.005	15	<0.005	11	14	<0.05
RMET		Total score	28	29	ns	30	ns	23	24	ns

Attribution style										
AIHQ										
	Ambiguous situations									
		Hostility score	1.6	1.6	ns	1.8	ns	2.6	1.6	<0.05
		Attribution of responsibility	1.87	2.3	ns	3.33	<0.005	3	2.87	ns
		Aggression score	1	1.6	<0.05	1.4	0.07	2	1.6	0.08
	Intentional situations									
		Hostility score	–	–	–	–	–	3.2	2.8	ns
		Attribution of responsibility	–	–	–	–	–	4.6	4.93	ns
		Aggression score	–	–	–	–	–	2.8	2.4	ns
	Accidental situations									
		Hostility score	–	–	–	–	–	1.4	1	<.005
		Attribution of responsibility	–	–	–	–	–	2	1.6	ns
		Aggression score	–	–	–	–	–	2.4	1.6	ns

Social perception and knowledge										
PerSo										
		Total interpretation score	18/24	22/24	–	21/24	–	17/24	22/24	–

Empathy										
EQ										
		Total score	39	45	ns	36	ns	–	–	–
QCAE										
		Total cognitive	–	–	–	–		56/76	54/76	–
		Total affective	–	–	–	–		39/48	37/48	–

*ns, not significant*.

Results globally corroborated previous conclusions. Damien’s assessment highlighted substantially enhanced scores in all ToM tests. The improvement was stable – and even increased – 9 months after the end of the therapy. We also observed improved facial emotion recognition after the therapy and at follow-up, even if the results did not reach statistical significance. Damien was also able to correctly recognize facial expressions with a lower level of intensity. Scores on the attribution bias test emphasized the evolution of scores throughout the treatment, but results were still close to those expected in controls. We also observed improved social perception and knowledge scores. His describing images depicting social situations was richer and his interpreting interactions between characters was more accurate at the end of the treatment.

Robin’s results also corroborated the conclusion of the previous the single-case study. We observed substantially enhanced ToM scores. Attributional style was one of the central target components of the therapy, and we noted a very positive and significant evolution of the AIHQ scores (61). Robin’s explanations of social situations were more qualified for both accidental and ambiguous situations. He was then able to submit several hypotheses to explain the potential causes of specific situations with different kinds of attributions – internal, external, and situational. We also noted improved facial emotion recognition measured with the TREF (57) after the therapy, although the results were not statistically significant.

Damien subjectively reported to have made progress in social functioning. He has recovered his previous circle of friends and meets them regularly. He is more comfortable during family reunions and his parents reported the same improvement. Damien seems to be more flexible during social interactions and said he was more able to adapt his behavior to the social context. At the end of the treatment, Robin reported the subjective feeling of being more comfortable in social situations. He explained he was less overwhelmed by negative interpretations in social interactions and was able to communicate with others more easily.

To assess the objective evolution of social functioning, the scales used before the therapy were repeated at the end of the treatment, and 9 months later in Damien’s case. The results are presented in Table 4.

**Table 4:** **Pre-, post-, and follow-up comparisons of functional assessments for patients A and B**.

		Patient A					Patient B		
		Pre-therapy scores	Post-therapy scores	*p* (pre vs. post)	Follow-up scores	*p* (pre vs. follow-up)	Pre-therapy scores	Post-therapy scores	*p* (pre vs. post)
SERS									
	Total	+5	+17	ns	+4	ns	−3	+3	ns
	Negative self-esteem	−34	−28	–	−29	–	−34	−34	–
	Positive self-esteem	+39	+45	–	+33	–	+31	+37	–
WEMWBS									
	Total	50	54	ns	49	ns	43	46	ns
EAS									
	Total	42	28	0.09	17	<0.05	–	–	
	Personal care	2	2	ns	2	ns	–	–	
	Daily life management	12	9	ns	8	ns	–	–	
	Resources management	6	6	ns	2	ns	–	–	
	External relationships	9	4	ns	0	<0.05	–	–	
	Emotional life and social relationships	13	7	0.07	5	ns	–	–	

A non-significant improvement of Damien’s self-esteem was reported at the end of the treatment, but 9 months later the SERS total score (47) came back to its initial level. Damien’s mental well-being, measured with the WEMWBS (48), remained stable throughout the treatment. We observed a general trend to improvement in daily social functioning assessed with the *EAS* (49) at the end of the therapy. The most impacted sub–score was “emotional life and social relationships.” Nine months after the end of the treatment, Damien’s total score decreased significantly compared to his initial score. All sub-scores decreased; especially the “external relationships” sub-score changed significantly. Finally, we noted decreasing PANSS total score of psychotic symptoms throughout the treatment, from 76 at the beginning to 72 at the end and 62 nine months after. This improvement seems to show in Damien’s behavior. Robin’s scores on the scales selected to assess self-esteem [SERS (47)] and mental well-being [WEMWBS (48)] remained stable, as did symptoms measured with the PANSS (46).

## Discussion

The main objective of these two single-case studies was to assess the impact of the RC2S program on social cognitive impairments in two patients with schizophrenia. We will now discuss the results in the light of the literature on the cognitive remediation of social cognition.

### RC2S Program Efficiency

RC2S was developed as a comprehensive social cognitive remediation program. It is an individualized treatment designed to adapt the therapy to the specific social cognition difficulties of each patient while taking into account preserved cognitive processes. The two single-case studies highlighted improvement in both patients’ abilities in the targeted processes, i.e., ToM in Damien’s case, and ToM and attributional style in Robin’s case. These improvements were emphasized through SBLs during the studies and social cognitive assessments after the therapy. Damien showed enhanced ToM abilities. The strategies developed during the treatment, such as analyzing context and behavior to make assumptions about another person’s mental state, also allowed him to progress in tasks involving social perception processes. As for Robin, we noted normalized scores on the attribution bias test and significantly improved ToM at the end of the therapy. Our two patients seemed to have developed efficient alternative strategies to compensate for their difficulties in specific social cognition domains. Furthermore, the UsBL, involving verbal memory processes that were untargeted by cognitive remediation therapy, remained stable throughout the treatment. This suggests that the results were not due to a general improvement of cognitive functioning.

Moreover, the generalization of the positive impacts of the RC2S therapy was also highlighted by the results for the IBLs and the social cognitive assessment at the end of the therapy. For each patient, IBLs focused on the recognition of basic and complex emotions on the one hand, and the understanding of others’ behaviors through social intelligence on the other. The performances on the two IBLs significantly improved for both patients by the end of the therapy. These results suggest that the RC2S program has a positive impact on social cognitive processes close to those targeted by the therapy, even if this enhancement is still weak in the long run. The social cognitive assessments performed at the end of the therapy also showed improved untargeted processes. Actually, even if such improvements were not statistically significant, the patients’ scores at the end of the therapy (and 9 months later in Damien’s case) were similar to those observed in the general healthy population. This provides additional arguments supporting RC2S as a comprehensive program that is able to target any component of social cognition, and focus on one process or another depending on the patient’s profile.

Concerning the transfer of the impacts of the therapy to daily life, the two patients reported subjective benefits on social functioning. Damien has renewed contact with his friends and has been participating more in group activities such as sports. Robin declared it was easier for him to start and maintain conversations with others. However, these subjective impacts were not confirmed by the scales assessing self-esteem and mental well-being. Only the *EAS* (49) highlighted significantly enhanced daily social functioning in Damien’s case 9 months after the end of the therapy. We were unfortunately not able to test Robin with the *EAS*; therefore, we could not conclude on the transfer of RC2S benefits to daily life. In addition, at least another criticism might explain these results. The measures assessing that social functioning may have been too different from the aspects of daily life practiced during the therapy. The analysis of the empirical literature on the impact of social cognitive remediation on functional outcomes indeed emphasizes that most studies assess social functioning with measures based on role-plays or real interactions, such as the Social Skills Performance Assessment (SSPA) (73). Such evaluation tools are clearly more adapted to reflect the patients’ achievements in social functioning than the questionnaires used in our single-case studies.

### RC2S Program in the Field of Social Cognitive Remediation

As discussed in the first part of this paper, the few meta-analyses assessing social cognitive remediation in schizophrenia emphasized promising results (32, 33), even if some conclusions seem to be less clear-cut when the data from social cognitive therapy patients are compared to controls (74). Some components of social cognition, especially basic processes, such as emotion recognition and social perception, clearly appear to benefit from social cognitive remediation. ToM seems to be positively impacted, but the long-term maintenance of benefits and the replication of results have only been occasionally studied. Finally, it seems more difficult to have an impact on higher-order social cognitive functions, such as attributional style, for which very few studies report significant improvements.

The results of the two single-case studies presented in this paper partly support the data from the literature. Indeed, we observed a normalization of the capacity to recognize facial emotions, even though this component was not specifically targeted by the therapy. This result confirms that basic processes of social cognition can be definitely improved by cognitive remediation. We also observed enhanced ToM abilities for both patients. This component of social cognition was a therapeutic target for both participants, who have significantly improved their performances on SBLs and pre- and post-therapy tests. These results support the potential for ToM improvement through social cognitive remediation. Lastly, the single-case study carried out on Robin (patient B), who presented with attributional style impairments, emphasized that social cognitive remediation could have an impact on higher-order social cognitive functions. Indeed, Robin improved his performances both on the attributional style SBL and the attribution bias test. The impact of cognitive remediation on attributional style is scarce in the literature but our results are consistent with those obtained with the Social Cognition and Interaction Training program (SCIT) (75). Researchers showed significant attributional style improvement in several studies evaluating the impact of SCIT on social cognition (75–79). We might actually consider that our results are linked to the strategies used in our study. During Robin’s therapy, the strategies developed to enhance attributional style were quite similar to those in the SCIT program, namely the detachment from the patient’s initial interpretations of specific social situations by focusing on the facts that provided arguments for alternative interpretations. Moreover, in their review, Fiszdon and Reddy (33) suggested that the limited success of social cognitive remediation on higher-order functions might be due to limited opportunities for the patients to practice skills. In RC2S, we chose to bypass this difficulty by using simulation scenes as a primary transfer step to test and adapt the strategies developed during paper-and-pencil tasks to real life-like situations. The gap between training and real life is, therefore, smaller than in other social cognitive remediation programs. This could be one of the strongest assets of the RC2S program.

### Relevance of an Individual and Computerized Therapy

As described in the introductive part, the use of computerized programs in the field of social cognition is scarce and most therapies are designed for groups, based on the assumption that improved social skills abilities are essentially favored by therapies involving social situations. However, group therapies present a few disadvantages: since the exercises are standardized for the entire group and each participant’s specific profile is not taken into account, they neither include adapted strategies to compensate for or restore cognitive deficits, nor personalized exercises aiming to transfer strategies to daily life (80). Furthermore, computerized programs provide advantages, both to adapt the cognitive remediation therapy to patients’ specific impairments and abilities, but also in term of impacts on cerebral functioning. Actually, according to a meta-analytic study assessing the efficacy and specificity of computer-assisted cognitive remediation in schizophrenia prolonged multimedia stimulation favor neural plasticity (81), which is a central concept of cognitive remediation.

Even if computerized programs are not widespread in social cognitive remediation, these techniques, especially those involving simulation or virtual reality, are already used in the field of psychiatry and neuropsychology for treating specific phobias, post-traumatic stress disorder, or attention-deficit disorder in children (82). More recently, virtual-reality systems has been used for social skills training of people with schizophrenia (40–43) and authors found these technologies are particularly good at improving conversational skills and assertiveness, but also negative symptoms, psychopathology, and social functioning. Our results confirmed previous data and demonstrated the interest of a computerized and individualized program to improve specific social cognitive functions. Virtual-reality applications seem to be also advantageous in terms of enhancing motivation for therapy. Based on our experience, computerized tasks seem to be actually very motivating for patients, and particularly for young ones. This result is interesting because recent models of cognition suggest that motivation is a core component of the relationship between social functioning and cognition in schizophrenia (83).

### Improvement of the RC2S Program and Validation Study

These single-case studies also paved the way for enhancing the program. Actually, we have developed various sets of photos and videos to complete the simulation scenes and potentiate the therapeutic impacts on social cognition and functioning. The combination is called RC2S+.

Two sets of photos were developed with the help of professional actors: one set was designed to work on facial and postural emotions, including basic and more complex emotions, such as boredom, pride, shame, or seduction with several levels of intensity; the other set depicts situations involving either one person whose emotions or mental state can be inferred thanks to contextual information, or several people in daily-life activities. This material allows for the training of ToM and social perception abilities and is also a valuable basis for discussions around attributional style. We have also developed videos that specifically target attributional style by presenting both ambiguous interactions and archetypal social situations. All this additional material can be used during paper-and-pencil tasks and allows the therapist to select specific items according to the patient’s profile and interests. Moreover, in order to enhance massed practice, we have decided to split up cognitive remediation sessions into two 1-h sessions per week: a paper-and-pencil session to develop strategies and a computerized session to use strategies in simulation scenes. Finally, we are also evaluating the possibility of a mixed individual/group therapy with individual paper-and-pencil and small-group computerized sessions to favor both the exchange of strategies and discussions between patients, depending on their points of view on the interactions between Tom and other characters.

To conclude, the RC2S program is a new promising tool for reducing social cognitive impairment in schizophrenia. RC2S was also used in other populations presenting with the same kind of deficits, such as a young man with schizoid personality disorder, a woman with 22q11 deletion syndrome, and a patient with Prader–Willi syndrome without intellectual disability (personal data). All the results were positive and corroborated the conclusions of the two single-case studies reported in this paper. We are currently preparing a randomized controlled trial to establish the validity of the RC2S program in schizophrenia. The study will be conducted in 2016 by several teams in France and aims to assess the impact of RC2S on attributional style compared with cognitive behavioral therapy focusing on positive symptoms.

## Author Contributions

EP and NF both contributed to the conception of the RC2S program. The design of the studies has been proposed by EP, such as the acquisition and analysis. EP and NF both participated in the interpretation of data for the work. EP drafted the paper, and NF and EP both approved the final version.

## Conflict of Interest Statement

The research was conducted in the absence of any commercial or financial relationship that could be considered as a potential conflict of interest.

## Footnotes

^1^http://www.remediation-cognitive.org/documents
